# Giant Thoracic Meningioma: Missed Diagnosis and Challenging Management in a Resource-Limited Setting

**DOI:** 10.7759/cureus.104315

**Published:** 2026-02-26

**Authors:** Gerald Musa, Aaron Munkondya, Lukulula E Mwanza, Sandford Sumaili, Mwaba Nambela, Davies Chiwaya, Chifundo Daka, Kabongo Ngoy, Keith Simfukwe, Misa Funjika, Carlos Castillo-Rangel, Gervith Reyes Soto, Manuel De Jesus Encarnacion Ramirez, Nicola Montemurro, Justo Banda

**Affiliations:** 1 Neurosurgery, Ndola Teaching Hospital, Ndola, ZMB; 2 Neurosurgery, University Teaching Hospital, Lusaka, ZMB; 3 Neurosurgery, Maina Soko Medical Center, Lusaka, ZMB; 4 Ophthalmology, Ndola Teaching Hospital, Ndola, ZMB; 5 Neurosurgery, Hospital Regional “1° de Octubre” del Instituto de Seguridad y Servicios Sociales de los Trabajadores del Estado (ISSSTE), Mexico City, MEX; 6 Neurosurgical Oncology, Mexico National Cancer Institute, Tlalpan, MEX; 7 Neurosurgery, Traumatology Hospital of Azua, Azua, DOM; 8 Neurosurgery, Azienda Ospedaliero Universitaria Pisana (AOUP), Pisa, ITA; 9 Urology, Ndola Teaching Hospital, Ndola, ZMB

**Keywords:** cerebrospinal fluid leak, delayed diagnosis, intradural extramedullary tumor, resource-limited neurosurgery, spinal meningioma

## Abstract

Intradural extramedullary spinal tumors are surgically treatable lesions, but delayed diagnosis, particularly in low-resource settings, can result in irreversible neurological deficits. We present a case of a 37-year-old woman with a five-year history of progressive paraplegia due to a thoracic T4-T5 intradural extramedullary tumor, whose diagnosis and management were significantly delayed due to system-level limitations. This case illustrates the severe consequences of missed or delayed diagnosis of surgically treatable spinal tumors in resource-limited settings. Early MRI interpretation by trained specialists, timely neurosurgical referral, and availability of surgical adjuncts such as dural sealants are essential to prevent avoidable morbidity.

## Introduction

Intradural extramedullary (IDEM) spinal tumors, principally meningiomas and nerve-sheath tumors (schwannomas/neurofibromas), account for a substantial proportion of primary spinal neoplasms and are a frequent cause of progressive myelopathy when they occur in the thoracic canal [1,2]. Because IDEM lesions are often slow-growing, clinical onsets may be insidious, as patients typically present with sensory change, axial or radicular pain, and progressive motor weakness. When recognized early, gross total resection is often achievable and associated with favorable neurologic recovery and long-term disease control [1-4]. However, the degree of postoperative neurological improvement correlates strongly with the magnitude and duration of preoperative cord compression and the severity of deficits at presentation; prolonged compression portends worse motor recovery despite technically successful tumor removal [5-7].

In high-income settings, early MRI access and multidisciplinary care pathways enable prompt diagnosis and expedited neurosurgical intervention, optimizing functional outcomes [1,3]. In contrast, in low- and middle-income countries (LMICs), diagnostic delays are common and are driven by limited MRI availability, suboptimal image interpretation, lack of neurosurgical specialists, and logistical barriers to referral, factors that collectively increase the risk of irreversible spinal cord injury [8-10]. Moreover, perioperative complications, such as cerebrospinal fluid (CSF) leakage and wound infection, remain important causes of morbidity after IDEM surgery; the choice of closure technique and adjuncts (suture technique, sealants, drains) influences leak rates, although the evidence for routine prophylactic sealant use remains mixed [11-13]. We present a case of a young woman with a five-year history of progressive paraparesis up to paraplegia due to a thoracic intradural extramedullary tumor, whose diagnosis and management were significantly delayed due to system-level limitations.

## Case presentation

A 37-year-old woman presented to our tertiary hospital with a five-year history of progressive neurological decline. She was assessed and referred to the neurosurgical unit. Her symptoms began insidiously with unilateral paresthesia on the left side of the trunk and lower limb, which gradually progressed to involve both lower extremities. Initial evaluation at a local hospital resulted in empirical treatment with multivitamin supplementation, without symptomatic improvement. Over the next two years, her gait progressively deteriorated until she became wheelchair-dependent, and by three years prior to presentation, she was completely bedridden.

Two years before referral, a magnetic resonance imaging (MRI) study was reportedly performed at the local facility and interpreted as normal; however, the images were unavailable for review. During this period of immobility, she developed a sacral pressure ulcer that was managed with regular wound care. She had never been evaluated by a neurosurgeon. Upon referral to our center, she was alert, well-nourished, and afebrile. Neurological examination revealed spastic paraplegia, marked bilateral lower-limb muscle atrophy, and absence of all sensory modalities below the T5 dermatome. The patient had been catheterized. A healing grade IV sacral decubitus ulcer was present.

**Figure 1:**
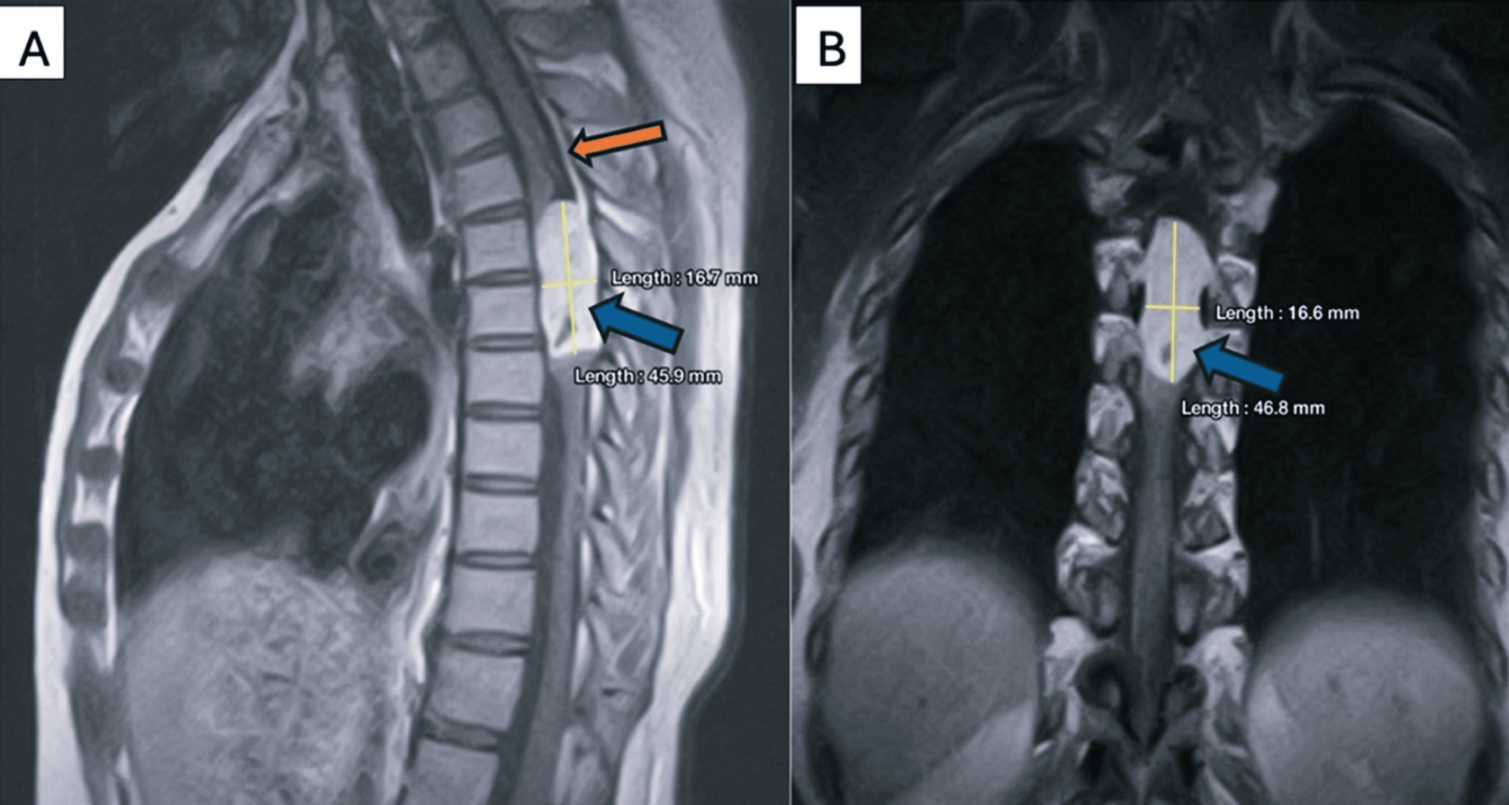
Thoracic MRI with gadolinium contrast. Sagittal (A) and coronal (B) views showing a homogeneously contrast-enhancing lesion over T4-T5 vertebrae (blue arrows) measuring 45.9×16.7×16.6 mm. Dural thickening (dural tail) is visible on the superior border of the tumor (orange arrow). The tumor fills the spinal canal completely, compressing the spinal cord.

An urgent contrast-enhanced MRI of the thoracic spine demonstrated a large intradural extramedullary mass at T4-T5, showing homogeneous postcontrast enhancement and causing near-complete obliteration of the spinal canal at that level. The spinal cord was severely compressed and poorly visualized (Figures 1A, 1B). Given the chronicity of symptoms and imaging findings, she was counseled extensively regarding prognosis. Surgical resection was planned.

Surgical procedure

**Figure 2:**
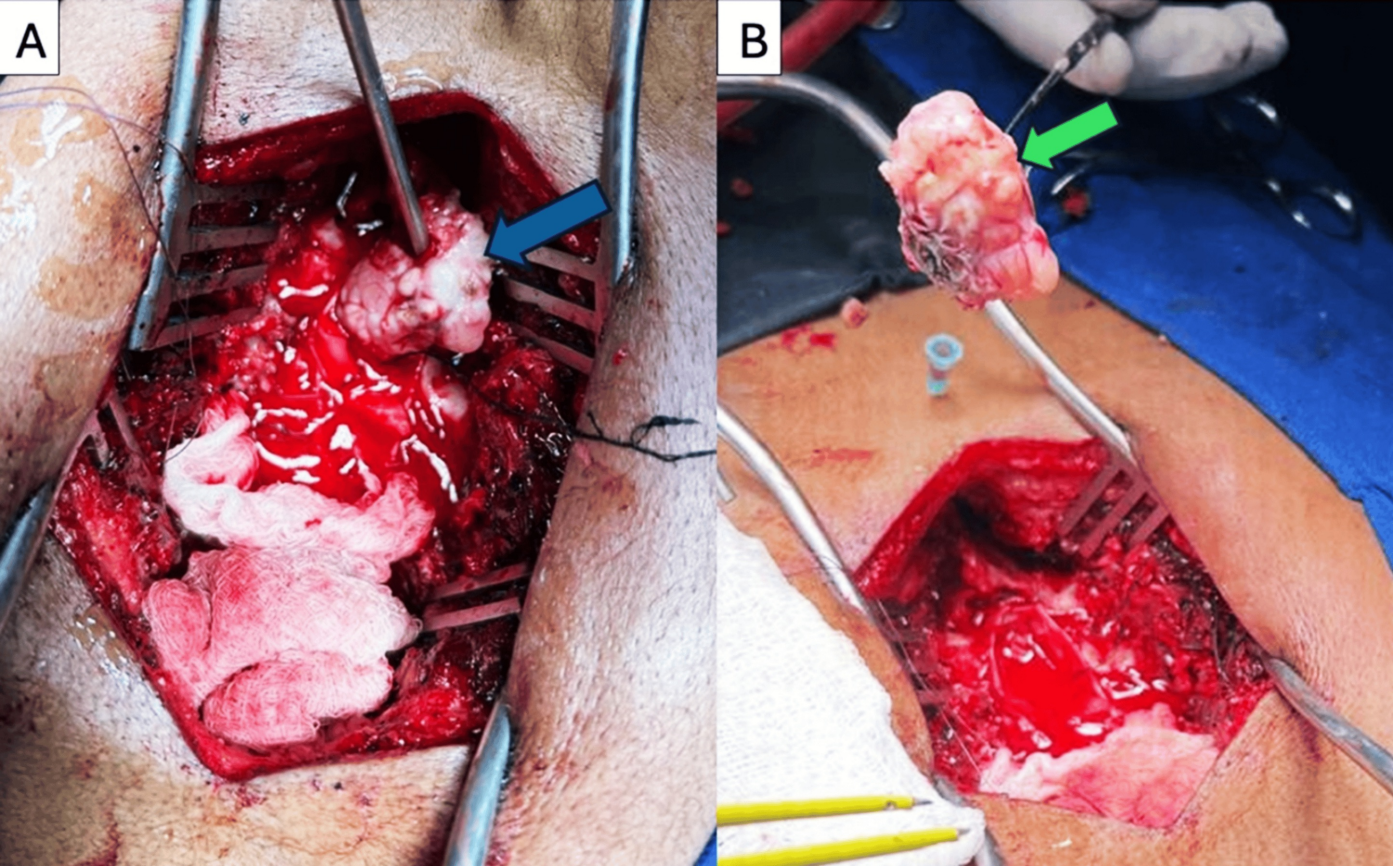
Intraoperative images showing the tumor during (A; blue arrow) and after resection (B; green arrow).

Under general anesthesia, the T4-T5 level was confirmed using intraoperative fluoroscopy. A midline incision was made from T3 to T6. Wide laminectomies of T4 and T5 were performed with preservation of the facet joints. The dura was tense, and the underlying mass was readily palpable. A midline durotomy was performed, extending cranially and caudally until normal dura margins were identified. A firm, light-gray intradural extramedullary tumor was visualized predominantly on the left side, displacing the thoracic spinal cord to the right. The tumor was devascularized and meticulously dissected from the spinal cord, preserving the arachnoid plane. Its dural attachment was coagulated, and the mass was removed en bloc, achieving a Simpson grade 2 resection (Figures 2A, 2B). No spinal fixation was required. The dura mater was closed with a waterproof monofilament suture. A subfascial drain was placed, and the wound was closed routinely. Prophylactic antibiotics were administered.

Postoperative course and surgical complications

**Figure 3:**
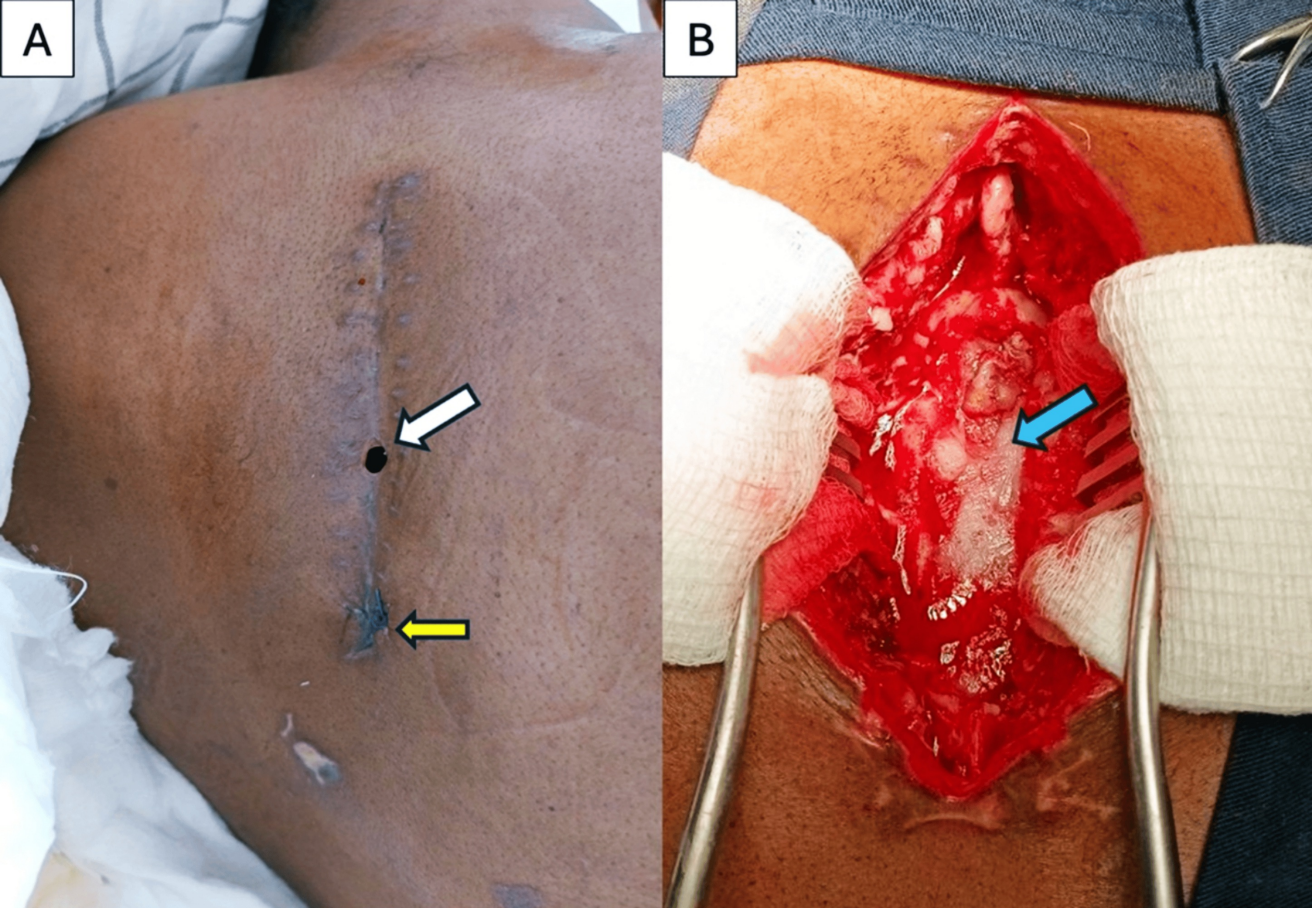
Postoperative course showing wound dehiscence and subsequent dural repair. (A) Wound on day 10 showing focal wound dehiscence caused by CSF leak (white arrow). A suture is visible at the caudal end of the scar (yellow arrow). (B) Intraoperative image during the second surgery showing dura glue applied over the dura after repair (blue arrow).

On postoperative day (POD) two, she developed severe positional headaches suggestive of cerebrospinal fluid (CSF) leakage. The drain was removed, but by POD four, the wound demonstrated CSF-mixed serosanguinous discharge. Conservative measures, including wound compression, daily dressing changes, and acetazolamide, offered minimal benefit, and headaches persisted. Wound culture grew *Klebsiella pneumoniae*, sensitive to meropenem. Antibiotics were changed accordingly to meropenem 1 g three times daily. Over the following days, the wound dehiscence worsened (Figures 3A, 3B). Due to the unavailability of dural sealant on site, definitive surgical repair was delayed by five days while the material was procured. During this period, conservative management and intravenous antibiotics were continued.

Post-discharge course, revision surgery, and outcome

On POD 14 from the initial tumor resection, the patient underwent re-exploration and dural repair. The previous incision was reopened, and approximately 150 mL of serosanguinous fluid was evacuated. The durotomy repair site was inspected, revealing a 2-4 mm tear along the previous suture line with active CSF egress. The defect was closed with interrupted 6-0 Prolene sutures without disturbing the intact portions of the initial repair. A Valsalva maneuver confirmed the absence of further leakage. Tisseel dural sealant was applied over the repair site. A closed-suction drain was placed, and a layered closure was performed. Postoperatively, she remained clinically stable and continued meropenem for 10 days. The drain was removed on POD six, and the wound healed without further complications.

At discharge (10 days after the second surgery), the patient demonstrated complete recovery of deep and superficial sensation in both lower limbs. Motor function remained absent, with sensory improvement from American Spinal Injury Association (ASIA) Impairment Scale A to ASIA B. She was referred to the physiotherapy department for long-term intensive neurorehabilitation. Histopathology showed meningioma grade 1.

## Discussion

This case typifies the biologic behavior and clinical consequences of thoracic intradural extramedullary (IDEM) tumors and underscores principles supported in existing literature. Thoracic meningiomas commonly present with progressive myelopathy due to the relatively narrow canal; early resection generally yields substantial motor and sensory recovery when intervention occurs before prolonged cord damage [1,2,4]. Multiple series report that gross total resection (Simpson I-III) is achievable in the majority of primary IDEM tumors and is associated with low recurrence and good functional outcomes [1,3,4]. Functional gains are most pronounced when surgery occurs early in the course of disease; several cohort studies and reviews demonstrate that shorter symptom duration predicts better motor recovery and ambulatory status postoperatively [5-7]. The patient in our report experienced a protracted five-year symptomatic period, consistent with literature that links long delays to diminished motor recovery despite tumor removal [6,7].

Perioperative complications, especially CSF leak and wound infection, are well documented after intradural spinal surgery and present a particular challenge in settings lacking prompt access to adjuncts (e.g., sealants, specific graft materials). Systematic reviews of dural sealants and closure techniques find mixed evidence as follows: sealants may reduce persistent leaks in select settings but do not uniformly substitute for meticulous watertight primary closure [11,12]. Management algorithms emphasize an escalation from conservative measures (positioning, local compression, acetazolamide, antibiotics when indicated) to definitive surgical repair when leaks persist, or a wound infection occurs [13,14]. Such circumstances are not uncommon in low- and middle-income countries (LMICs) surgical settings, where limited access to essential materials, supply-chain disruptions, and resource constraints can significantly affect procedural timing, infection control, and overall patient outcomes.

The impact of health-system factors on outcomes after spinal tumors is increasingly recognized. Analyses of neurosurgical capacity in low-resource environments highlight shortages of imaging, trained radiologists, neurosurgeons, perioperative nursing support, and essential consumables; these gaps lead to delayed referrals, suboptimal preoperative assessment, and increased complication rates [9,15]. Case series from diverse regions show that when patients present late, odds of achieving functional ambulation fall considerably; conversely, focused efforts to strengthen referral pathways and imaging review by spine-experienced clinicians correlate with earlier surgery and improved functional endpoints [10,16-21].

Practical lessons and strategies

For resource-constrained hospitals, pragmatic interventions can reduce similar harms. Training general radiologists and frontline clinicians to recognize red flags (progressive myelopathy, bilateral signs, and a sensory level) and to escalate to tertiary centers can compress diagnostic intervals. Teleradiology or remote MRI review by spine specialists provides high value when local expertise is limited. Ensuring an essential list of neurosurgical consumables (watertight suture materials, basic sealants, closed-suction drains, empirical broad-spectrum antibiotics for wound infection) and prearranged procurement pathways reduces treatment delays. Where sealants are unavailable, careful multilayered closure and use of lumbar CSF diversion (when feasible) are supported alternatives in guidance documents.

The unavailable initial MRI precluded assessment of whether the lesion was present but misread versus progressive growth after imaging - a distinction affecting interpretation of “missed diagnosis.” Comparative outcome data are primarily retrospective and heterogeneous in tumor histology, preoperative status, and follow-up, limiting precise prognostication for individual patients. Finally, many high-quality studies are conducted in high-resource centers; translating their protocols to LMIC contexts must consider feasibility and supply-chain constraints. Despite these limitations, the case is instructive because it concretely demonstrates how system-level failures (imaging interpretation, delayed neurosurgical referral, and shortages of simple consumables) combine to worsen neurologic outcomes, a theme echoed across recent global neurosurgery literature.

## Conclusions

This study highlights the vital importance of prompt recognition and timely referral in patients presenting with progressive myelopathy, emphasizing the need for early intervention to optimize clinical outcomes. In resource-limited settings, diagnostic errors, inadequate interpretation of imaging, and delayed access to neurosurgical care can lead to prolonged spinal cord compression and irreversible neurological deficits. Improving diagnostic pathways, strengthening radiology-neurosurgery collaboration, and ensuring the availability of basic operative resources, such as dural sealants, are key to improving outcomes for patients with spinal tumors.
